# Oclacitinib for the Treatment of Nasal Alar Arteriopathy in Two Dogs

**DOI:** 10.3390/ani16121915

**Published:** 2026-06-20

**Authors:** Katherine Bingham, Mara Kraenzlin, Dianne Kittrell, Beth Whitney, Andrew McGlinchey, Nina Shoulberg

**Affiliations:** 1 Department of Veterinary Internal Medicine, Shoreline Veterinary Referral and Emergency Center, Shelton, CT 06484, USA; katherine.bingham@vca.com (K.B.); dianne.kittrell@vca.com (D.K.); 2 Department of Veterinary Critical Care Medicine, Shoreline Veterinary Referral and Emergency Center, Shelton, CT 06484, USA; 3 Department of Veterinary Internal Medicine, Advanced Veterinary Specialists, Santa Barbara, CA 93101, USA; 4 Department of Veterinary Internal Medicine, Veterinary Referral and Emergency Center, Norwalk, CT 06854, USA; 5 Department of Veterinary Dermatology, Veterinary Referral and Emergency Center, Norwalk, CT 06854, USA

**Keywords:** dog, canine, nasal alar arteriopathy, oclacitinib, dermal arteritis of the nasal philtrum

## Abstract

Nasal alar arteriopathy (NAA) is a rare dermatologic condition in dogs characterized by ulcerative and potentially severe hemorrhagic lesions of the nasal alar fold. This condition has only previously been reported in German Shepherd Dogs. Achieving clinical remission can be challenging and typically involves a combination of surgical resection of the diseased tissue and immunosuppressive therapies. This report is the first to describe two cases of NAA in which clinical remission was initially achieved with oclacitinib alone. Both cases were presented to the Internal Medicine service with a complaint of unilateral recurrent epistaxis. The first case involved a 7-year-old German Shepherd dog. Clinical remission was first achieved with oclacitinib monotherapy. Relapse occurred following dose tapering, and remission was ultimately regained with dose reescalation of oclacitinib and the addition of cyclosporine. The second case involved a 3-year-old Poodle mix that achieved and maintained clinical remission with oclacitinib alone. These cases suggest that oclacitinib may be an effective and well-tolerated treatment option for NAA, offering an alternative to traditional systemic immunosuppressive therapies. However, additional treatment may be required in severe cases or during oclacitinib dose reduction. Further studies are needed to evaluate long-term outcomes and broader applicability.

## Simple Summary

Nasal alar arteriopathy (NAA) is a rare dermatologic condition in dogs that causes ulcerative lesions and the potential for severe bleeding from the nasal alar fold. Management of disease symptoms can be challenging and typically involves surgical resection or a combination of topical ointments and systemic immunosuppressives. This study is the first to report the initial successful use of sole oclacitinib therapy, a medication typically used to treat atopic dermatitis, to manage NAA in two dogs. While achieving clinical remission can be challenging and relapse can often occur, both dogs experienced significant improvement in their symptoms after starting treatment with oclacitinib alone. Oclacitinib is a well-tolerated medication with the rare potential for systemic side effects and may be an alternative treatment for patients that are not candidates for systemic immunosuppressives or topical medications.

## 1. Introduction

Dermal arteritis of the nasal philtrum (DANP) is a rare dermatologic disorder characterized by well-demarcated ulcerative lesions associated with varying degrees of arterial hemorrhage. The lesions are seemingly non-painful and non-pruritic, and arterial hemorrhage can range from mild and intermittent to severe [1,2,3,4,5,6]. In severe cases, emergency treatment to achieve hemostasis and administration of packed red blood cell transfusions may be warranted [5]. Histopathological findings of DANP include subendothelial proliferation of spindle cells within the deep dermal arteries and arterioles adjacent to the ulcer, deposition of extracellular matrix and variable degrees of inflammation [1]. This inflammation leads to intimal thickening and stenosis of the dermal arteries and arterioles, which can result in arterial necrosis, thrombosis and hemorrhage [5]. The etiology of the disease is unknown, but favorable responses to immunomodulatory therapy suggests an immune-mediated component [6]. 

The mainstay of treatment for DANP usually involves a combination of immunosuppressive and anti-inflammatory medications including oral prednisone, topical tacrolimus, doxycycline and niacinamide combinations, pentoxifylline, and cyclosporine. Surgical resection of the diseased tissue and ligation of feeding arteries has also been performed in dogs that are refractory to treatment [3]. Achieving full remission can be challenging and affected dogs may need to receive some degree of life-long treatment, even after surgical intervention [1,2,3,4,5].

While reports of disease affecting the nasal philtrum in DANP are widely documented, to the authors’ knowledge, histologically and grossly similar disease affecting the nasal alar in nasal alar arteriopathy (NAA) has only been reported in German Shepherd dogs [6]. In NAA, ulcerative lesions with the potential for arterial hemorrhage occur unilaterally on the nasal alar fold. In those affected with NAA, treatment to achieve partial or full remission has included topical tacrolimus, oral prednisone, cyclosporine, pentoxifylline, antimicrobials and doxycycline/niacinamide [6]. 

The purpose of this paper is to report two cases of NAA in a German Shepherd and Poodle mix dog that were both initially successfully treated with oclacitinib alone. Oclacitinib is FDA-approved for the treatment of atopic dermatitis but has more recently been reported to successfully treat numerous autoimmune dermatologic conditions including canine autoimmune subepidermal blistering dermatosis, pemphigus vulgaris, and chronic cutaneous lupus erythematosus variants [7,8]. Oclacitinib is a nonselective Janus kinase (JAK) inhibitor with preferential selection for JAK1. Inhibition of JAK1 downregulates the activation of inflammatory cytokines, some of which are involved in autoimmunity, such as IL-2, IL-15, IFN-α and IFN-y [7,8]. 

To the authors’ knowledge, there are no reports of oclacitinib having been used as the sole therapy to treat DANP or NAA previously. This case report is the first to explore whether treatment with oclacitinib may be successful in achieving clinical remission in NAA. One of the two cases in this report was a Poodle mix dog, demonstrating that NAA may occur in breeds other than German Shepherds. 

The use of a medication as clinically well-tolerated as oclacitinib to achieve clinical remission can now be considered in dogs where systemic immunosuppressive medications such as prednisone and cyclosporine are contraindicated.

## 2. Case Description

### 2.1. Case 1

A 7-year-old male neutered German Shepherd Dog weighing 45.2 kg was evaluated by the hospital’s Internal Medicine service for an approximately 2-year history of unilateral intermittent epistaxis. He had been receiving topical tacrolimus ointment (0.10% applied topically to the affected area q12h) for suspected discoid lupus; however, biopsies had not been obtained. The dog also received a tapering course of prednisone (0.88 mg/kg PO q12 × 5 days, 0.88 mg/kg PO q24h × 5 days, 0.88 mg/kg PO EOD for 2 doses). Complete blood count, chemistry panel, Accuplex and Fecal OPG were unremarkable (Antech Diagnostics, Middletown, CT, USA). Hemorrhagic episodes ranged in severity, but when severe bleeding occurred it was high-pressure and projectile. The dog was evaluated through an emergency clinic the week prior for severe epistaxis where hemostasis was reportedly achieved. 

On initial physical exam, there was a 4.0 mm ulcerative fissure on the right dorsal alar fold as well as moderate hyperkeratosis of the dorsal nasal planum. Rhinoscopy with the potential for nasal biopsies was recommended. 

**Figure 1:**
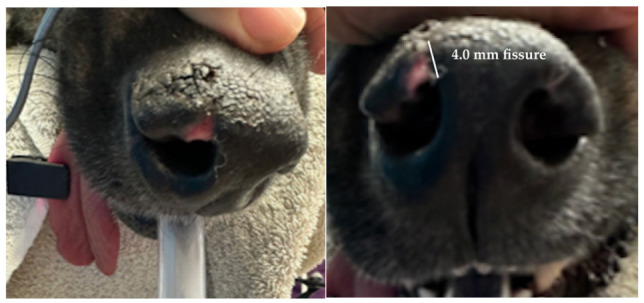
Images of the ulcerative fissure and hyperkeratosis of the right nasal alar fold.

The dog was anesthetized with diazepam (0.20 mg/kg IV) and butorphanol (0.2 mg/kg IV) and propofol IV to effect and maintained with isoflurane. Nasal turbinates appeared normal on rhinoscopy. An external nasal punch biopsy of the right and left dorsal alar fold was obtained (Figure 1). The dog was started on oclacitinib (0.53 mg/kg q12h) and Yunnan Baiyao (2 capsules PO q12h to be administered for 14 days) while awaiting biopsy results. Topical tacrolimus was discontinued. 

Histopathology results of the nasal planum revealed epidermal hyperplasia and variable parakeratotic hyperkeratosis in both samples (left and right nasal planum). In the left nasal planum, a dermal arteriopathy characterized by luminal stenosis, tunica intimal thickening, and karyorrhectic debris within the tunica media was present in a single mid-to-deep dermal arteriole. In the sample from the right nasal planum, a discrete, linear, and vertically oriented epidermal ulcer was present and accompanied by spongiosis, lymphocytic and neutrophilic exocytosis, and an equivocal focal capillary vasculitis. These findings were most consistent with nasal alar arteriopathy (NAA). 

Oclacitinib was initiated at 0.53 mg/kg PO q12h for 14 days, and then the dose was tapered to 0.53 mg/kg PO q24h. At revaluation 29 days after rhinoscopy and biopsy, the dose of oclacitinib was increased again to 0.53 mg/kg PO q12h due to the persistence of the nasal fissure. The dog had not experienced any hemorrhage from the ulcerative lesion at this time. It was advised to consider tapering the oclacitinib to q24h dosing if the nasal fissure improved and if hemorrhage had not recurred after 2–3 months of twice daily therapy. 

At follow-up 9 months after diagnosis and initiation of oclacitinib, the dog was asymptomatic with no episodes of epistaxis and the fissure had resolved. The dog had been maintained on oclacitinib (0.53 mg/kg PO q24h) for many months with no episodes of epistaxis.

Subsequently, 13 months after the initiation of oclacitinib, the dog was reevaluated due to the recurrence of an ulcerated fissure of the right nasal alar fold. No hemorrhagic events were reported. On physical examination there was a 1.0 cm ulceration of the dorsal aspect of the right alar fold and marked hyperkeratosis of the dorsal nasal planum. The dose of oclacitinib had been tapered by his primary care provider 3 months prior to this event from 0.53 mg/kg PO q24h to 0.35 mg/kg PO q24h with a gradual recurrence of the ulcerative lesion. The dog was started on twice-daily topical tacrolimus 0.10% ointment (generic) and the dose of oclacitinib was increased to 0.53 mg/kg PO q12h. 

Then, 2 months after the documented relapse, the dog was reevaluated. The ulcerative lesion had worsened and was 1.5 cm in diameter. There were still no reported hemorrhagic events. Topical tacrolimus had been discontinued due to the owner having difficulty with administration. Cyclosporine was prescribed (4.4 mg/kg PO q24h) and initiated 2 months after the relapse.

The dog was evaluated 3 months post relapse and was still receiving cyclosporine (4.4 mg/kg PO q24h) and oclacitinib (0.53 mg/kg PO q12h) with marked clinical improvement noted. There was a remaining 0.75 cm shallow ulcer of the right alar fold with moderate hyperkeratosis involving mostly the dorsal nasal planum. Continuing both medications at current doses was advised. 

The dog was last evaluated 6 months post relapse. The fissure had resolved with only a depigmented 5.0 mm scar on the dorsal aspect of the right alar fold remaining. At the time of this paper, the dog had not experienced any symptoms associated with relapse.

### 2.2. Case 2

**Figure 2:**
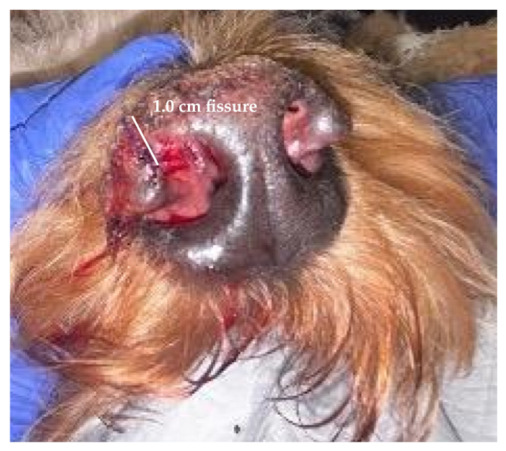
Image of the nasal fissure and ulcerative lesions of the right nasal alar fold.

A 3-year-old male neutered Poodle mix breed dog weighing 32.2 kg was evaluated by the hospital’s Internal Medicine service for a 3-week history of epistaxis that was characterized by recurrent hemorrhage from his right nasal planum. On physical exam, there was a 1.0 cm fissure of the right nasal alar fold with peripheral superficial ulcerative lesions (Figure 2). The dog had previously received a course of Clavamox (11.65 mg/kg PO q12h) with no improvement in symptoms. An over-the-counter liquid bandage was repeatedly applied but bleeding was persistent. The nasal planum was non-painful. Complete blood count and serum biochemical panels (Antech Diagnostics, Middletown, CT, USA) were performed and were unremarkable. 

**Figure 3:**
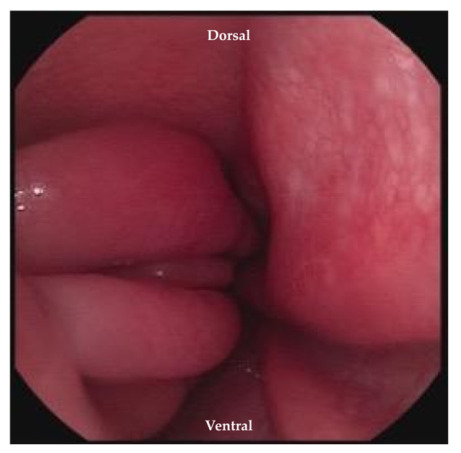
Normal-appearing nasal turbinates during rhinoscopic assessment.

The day after initial consultation, the dog was anesthetized with butorphanol (0.19 mg/kg IV), diazepam (0.19 mg/kg IV) and propofol IV to effect. Rhinoscopy was performed and the nasal turbinates appeared grossly normal (Figure 3). Three punch biopsies of the right nasal planum were obtained and dissolvable PDS 3-0 was used to close.

**Figure 4:**
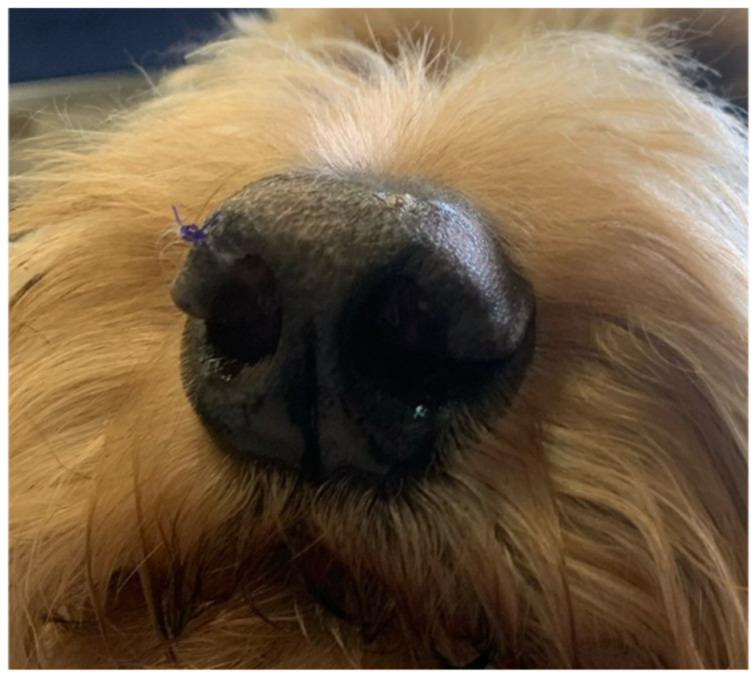
Resolution of the nasal fissure and superficial ulcerative lesions of the right nasal alar fold.

Biopsies revealed multifocal dermal arteriopathy with moderate epidermal hyperplasia and compact orthokeratotic hyperkeratosis consistent with nasal alar arteriopathy. The dog was started on oclacitinib (0.49 mg/kg q12h) for 30 days, and then the dose was decreased to 0.49 mg/kg PO q24h. Recheck examination was performed when the dog had been receiving once-daily oclacitinib for 10 days. There was full resolution of the nasal fissure and the ulcerative lesions at the time of recheck 40 days after initial presentation (Figure 4). Since the initiation of oclacitinib, there was no bleeding from the nasal alar lesion. The dog was clinically well and tolerating oclacitinib; 26 months after diagnosis, the dog has continued to receive 0.49 mg/kg PO q24h dosing and has maintained clinical remission on sole therapy.

## 3. Discussion

Achieving clinical remission in dogs with NAA can be challenging, with relapses and only partial remission often being documented in the histopathologically similar DANP [1,2,3,4,5]. 

The rarity of this disease leads to clinical confusion, as both cases in this report were initially evaluated for symptoms of suspected unilateral epistaxis. Without biopsy, NAA may be confused for differential diagnoses including lupus erythematosus, pemphigus vulgaris, contact hypersensitivity, neoplasia or trauma [1]. The first step to properly managing this disease requires achieving a proper diagnosis with histopathology. 

Considering that this is the first documented case of NAA affecting a Poodle mix, the degree to which this disease may affect a wide range of breeds is yet to be explored. Broader breed susceptibility cannot be concluded from a single non-German Shepherd case; however, maintaining a broad perspective when evaluating dogs with symptoms consistent with this disease is necessary to ensure that diagnosis and treatment is appropriate.

While reports of NAA are rare, treatment for the histopathologically similar DANP often involves a combination of antimicrobial, immunosuppressive and topical medications [1,2,3,4,5]. Challenges of ongoing treatment include the negative side effects of chronic systemic immunosuppressive medications including prednisone and cyclosporine. To date, NAA has only been described in German Shepherd dogs, and in DANP, large breed dogs are also overrepresented [2,6]. Large-breed dogs and those that are overconditioned may be more likely to experience negative prednisone-related side effects. Cyclosporine could be considered, but this medication may be cost-prohibitive and risks potential side effects. While topical tacrolimus is often utilized in DANP, its efficacy is largely limited by administration tolerance.

While the pathophysiology of NAA is unknown, the observed clinical response to prolonged topical and systemic immunomodulation supports an immune-mediated etiology [6]. In humans, JAK1 inhibitors have been shown to decrease multiple immune-response biomarkers that play key roles in autoimmune conditions such as rheumatoid arthritis [9]. It can be inferred that oclacitinib’s inhibition of JAK1, and the consequent downregulation of inflammatory cytokines (IL-2, IL-7, IL-9, IL-15, and IFN-γ), helps to control the inflammatory processes associated with immune-mediated conditions [8]. In the largest case series to date, immunohistochemical stains for T cells (CD3), histiocytes (IBA1) and neutrophils (myeloperoxidase) were negative in most specimens. This negative staining was suggested to reflect an “end-stage,” post-inflammatory lesion in which the initiating infiltrate may have already resolved [6]. Notably, IFN-γ signals predominantly through JAK1-dependent receptors and may drive keratinocyte apoptosis in oclacitinib-responsive canine hyperkeratotic erythema multiforme [8]. It is therefore possible that a similar IFN-γ-related mechanism underlies the response seen here, even without a histologically evident infiltrate, as IHC may miss the relevant effector populations in late-stage, post-inflammatory lesions.

This case report is novel and offers useful clinical insight, but it is necessarily limited by the small sample size and the absence of validation, as there was no control group, randomization, or standardized comparison with conventional therapies. Furthermore, although clinical remission was ultimately maintained on oclacitinib monotherapy until dose taper, the German Shepherd in Case 1 also received Yunnan Baiyao during the first 14 days post-biopsy to help ensure hemostasis would be achieved.

## 4. Conclusions

The use of oclacitinib to medically manage NAA provides an alternative treatment to those that may not be candidates for systemic immunosuppressive or topical medications. Oclacitinib is a systemically well-tolerated medication. Dosing of oclacitinib in these cases closely followed the FDA-approved recommendations for atopic dermatitis (0.4–0.6 mg/kg PO q12h for 14 days with a recommended taper to 0.4–0.6 mg/kg PO q24h long-term). The dog that relapsed while receiving oclacitinib monotherapy did so after tapering to 0.35 mg/kg PO q24h, which is below the recommended dose. Similar to cases described with DANP, multimodal therapy is often required, and the use of oclacitinib for treatment alone may not always be sufficient in more severe cases. Dosing of oclacitinib at the higher end of the dosing spectrum (>0.5 mg/kg/day) was needed in both cases in this report to achieve clinical remission. 

Long-term outcomes, ideally evaluated in prospective studies, are needed to determine the efficacy of treatment with oclacitinib for NAA. Considering the clinical and histological similarities that DANP shares with NAA, treatment of DANP with oclacitinib should also be considered and explored.

## Data Availability

The data presented in this study are available on request from the corresponding author. The data are not publicly available due to privacy restrictions related to client-owned animals.

## Abbreviations

The following abbreviations are used in this manuscript:

NAA	Nasal Alar Arteriopathy
DANP	Dermal Arteritis of the Nasal Philtrum
JAK	Janus Kinase
IL	Interleukin
IFN	Interferon
IV	Intravenous
